# Effects of intravesical BCG maintenance therapy duration on recurrence rate in high-risk non-muscle invasive bladder cancer (NMIBC): Systematic review and network meta-analysis according to EAU COVID-19 recommendations

**DOI:** 10.1371/journal.pone.0273733

**Published:** 2022-09-08

**Authors:** Young Joon Moon, Kang Su Cho, Jae Yong Jeong, Doo Yong Chung, Dong Hyuk Kang, Hae Do Jung, Joo Yong Lee

**Affiliations:** 1 Department of Urology, Severance Hospital, Urological Science Institute, Yonsei University College of Medicine, Seoul, Korea; 2 Department of Urology, Gangnam Severance Hospital, Urological Science Institute, Yonsei University College of Medicine, Seoul, Korea; 3 Department of Urology, Inha University College of Medicine, Incheon, Korea; 4 Department of Urology, Inje University Ilsan Paik Hospital, Inje University College of Medicine, Goyang, Korea; 5 Center of Evidence Based Medicine, Institute of Convergence Science, Yonsei University, Seoul, Korea; UNIBA: Universita degli Studi di Bari Aldo Moro, ITALY

## Abstract

**Purpose:**

During the coronavirus disease 2019 (COVID-19) pandemic, the European Association of Urology (EAU) recommended that courses of intravesical bacillus Calmette-Guérin (BCG) therapy lasting more than 1 year could be safely terminated for patients with high-risk non-muscle-invasive bladder cancer (NMIBC). Thus, we conducted a systematic review and network meta-analysis according to EAU’s COVID-19 recommendations.

**Materials and methods:**

A systematic review was performed following the Preferred Reporting Items for Systematic Review and Meta-Analysis guidelines. We conducted a network meta-analysis of recurrence rate in patients with NMIBC receiving induction therapy (M0) and those receiving maintenance therapy lasting 1 year (M1) and more than 1 year (M2).

**Results:**

Nineteen studies of 3,957 patients were included for the network meta-analysis. In a node-split forest plot using Bayesian Markov Chain Monte Carlo (MCMC) modeling, there were no differences between the M1 and M2 groups in recurrence rate [odds ratio (OR) 0.95 (0.73–1.2)]. However, recurrence rate in the M0 group was higher than that in the M1 [OR 1.9 (1.5–2.5)] and M2 [OR 2.0 (1.7–2.4)] groups. P-score tests using frequentist inference to rank the treatments in the network demonstrated that the therapy used in the M2 group (P-score 0.8701) was superior to that used in the M1 (P-score 0.6299) and M0 groups (P-score 0). In rank-probability tests using MCMC modeling, the M2 group showed the highest rank, followed by the M1 and M0 groups.

**Conclusion:**

In the network meta-analysis, there were no differences between those receiving BCG maintenance therapies in terms of recurrence rate. In the rank tests, therapy lasting more than 1-year appears to be most effective. During the COVID-19 pandemic, 1-year maintenance therapy can be used, but after the COVID-19 pandemic, therapy lasting more than 1-year could be beneficial.

## Introduction

Bladder cancer is the 10^th^ most common cancer in the world. The incidence of bladder cancer is increasing globally [1]. Approximately 75–85% of patients have the non-muscle invasive bladder cancer (NMIBC) type [2]. The treatment of choice for NMIBC is transurethral resection of the bladder tumor (TURBT) [3]. Features of NMIBC include a high recurrence rate after TURBT and the potential risk of progression to muscle-invasive disease [4]. Bacillus Calmette-Guérin (BCG) immunotherapy is considered the most effective adjuvant treatment to prevent recurrence and progression of high-risk NMIBC after TURBT [5].

BCG is a vaccine against tuberculosis that has been used as an immunotherapy for bladder cancer for more than 40 years [6]. In particular, three-quarters of early diagnosed bladder cancers are NMIBC, which is characterized by a high recurrence rate. Reduction of disease recurrence and prevention of progression to muscle-invasive disease are important considerations in NMIBC management [4]. In this aspect, intravesical BCG immunotherapy has been used as the backbone of adjuvant therapy after TURBT in patients with NMIBC [7].

Although the European Association of Urology (EAU)’s guidelines recommend 3-week instillations at 3, 6, 12, 18, 24, 30, and 36 months based on European Organization for Research and Treatment of Cancer data, the optimal duration of maintenance BCG is still unknown [8].

The coronavirus disease 2019, also known as COVID-19, has spread around the world, and the World Health Organization officially declared it a pandemic on March 11, 2020. Healthcare has been severely impacted, and urology practices have also been affected by the COVID-19 pandemic [9]. According to EAU’s COVID-19 recommendations, EAU’s NMIBC panel recommends 1-year intravesical BCG maintenance immunotherapy in patients with high-risk NMIBC [10]. Therefore, the purpose of this study was to determine the appropriate duration of BCG maintenance therapy during the COVID-19 pandemic since 2020.

## Materials and methods

### Inclusion criteria

We defined study eligibility following the Preferred Reporting Items for Systematic Review and Meta-Analysis (PRISMA) guidelines (S1 Table) [11]. The patient population was histologically confirmed to be NMIBC positive in tissue collected after TURBT. The intervention was intravesical BCG immunotherapy. The comparator was duration. The outcome was recurrence rate. The study design was a systematic review and meta-analysis. Patients were categorized into three groups: induction BCG therapy only (M0), 1-year BCG maintenance therapy (M1), and BCG maintenance therapy lasting more than 1 year (M2). Patients in the BCG maintenance therapy groups (M1 and M2) received induction BCG therapy followed by regular BCG maintenance therapy for at least 1 year, while patients in the M0 group received induction BCG therapy only. Any strain or dose of BCG was considered appropriate. We conducted this study based on the standard PRISMA guidelines [12].

### Search strategy

Literature searches for all publications prior to September 31, 2021 were carried out using PubMed and EMBASE. The following medical subject headings terms and keywords were used for the search: “urinary bladder neoplasms,” “urothelial carcinoma of bladder,” “transitional cell carcinoma of bladder,”“bladder carcinoma,” “bladder cancer,” “BCG,” “Bacillus Calmette-Guérin,” and “maintenance.”

### Data extraction

Two researchers (YJM and JYJ) screened the titles and abstracts of articles that were independently identified by the search strategy to exclude irrelevant studies. They also evaluated the full text of the articles to find potentially related articles. They extracted the most relevant articles in each study. Disagreements were solved by debate among the researchers until a consensus was reached.

### Quality assessment for studies

In the case of randomized controlled trials (RCTs), the Cochrane Risk of Bias tool was used, and in the case of nonrandomized studies, the methodological index for nonrandomized studies (MINORS) was used. Quality of evidence grading was performed using the Scottish Intercollegiate Guidelines Network (SIGN) checklist, consisting of various types of research. The quality assessment was conducted independently by our researchers.

### Heterogeneity tests

The Q statistic and Higgins’ I^2^ statistic were used for evaluations of study heterogeneity. Higgins’ I^2^ was calculated as follows:

$$I^{2}=\frac{Q-df}{Q}\times 100\%$$

where “Q” is Cochran’s heterogeneity statistic and “df” is the degrees of freedom. When the *P* value was less than 0.10, heterogeneity was considered significant. If evidence of heterogeneity existed, the data were analyzed using a random-effects model. Studies in which positive results had been confirmed were assessed with a pooled specificity using 95% confidence intervals (CIs).

### Statistical analysis

The primary outcome was tumor recurrence, which was measured using the odds ratio (OR) with 95% CIs. All statistical analyses were performed with R software (version 4.1.2, R Foundation for Statistical Computing, Vienna, Austria; http://www.r-project.org) and with the associated meta, netmeta, pcnetmeta, and gemtc packages for pairwise and network meta-analyses. This systematic review is registered in PROSPERO, CRD 42021291265.

## Results

### Eligible studies

**Fig 1:**
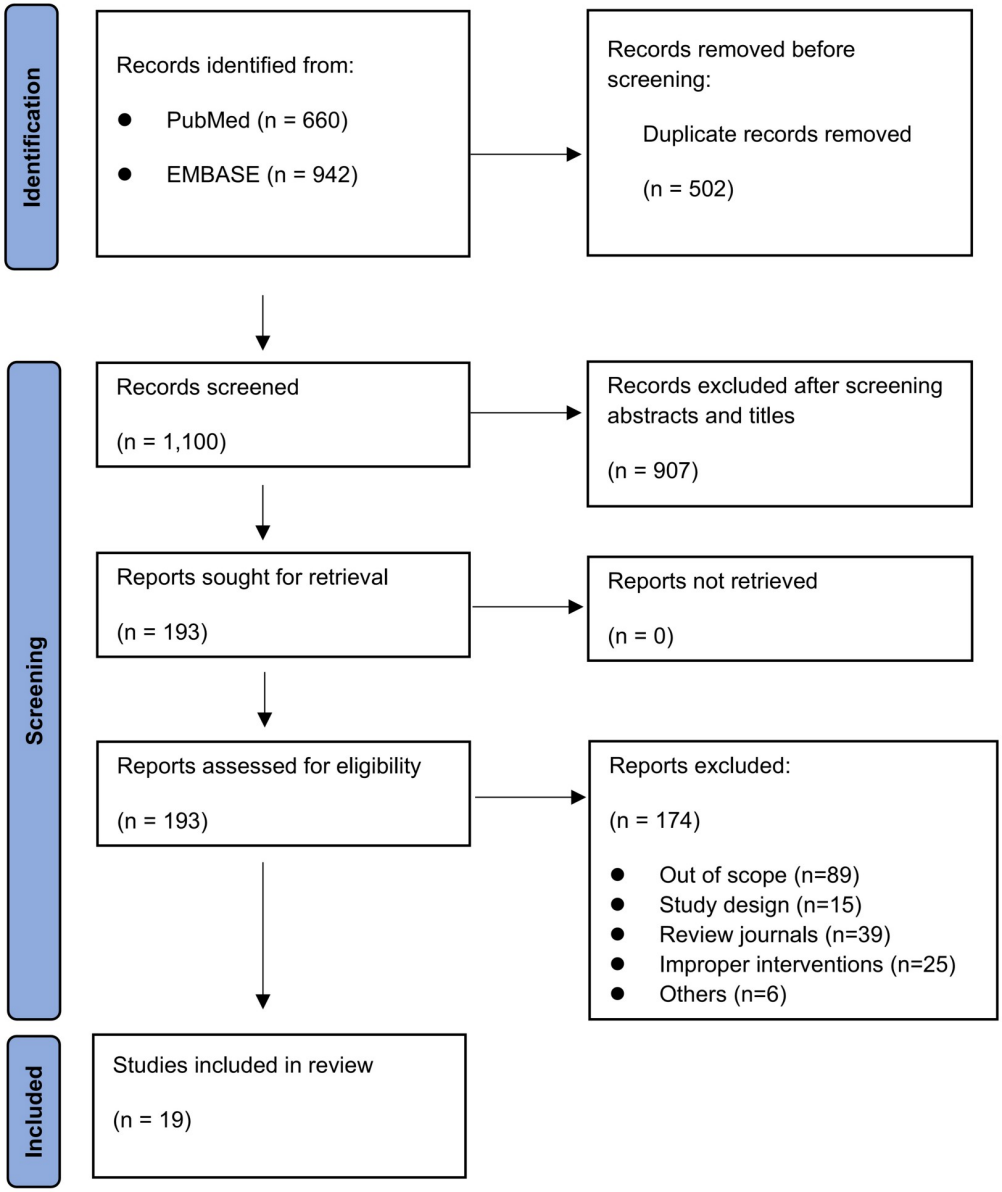
Study flow chart.

A total of 1,602 articles were verified by the initial database search. Of these, 1,409 articles were excluded: 502 were duplicate publications and 907 were excluded after reviewing the abstracts. A total of 193 articles were selected for full text evaluation. Further review excluded 174 articles because they were not relevant to the analysis: 89 were out of scope; 15 had a questionable study design; 39 were in review journals; 25 cited improper interventions; and 6 were excluded due to other causes. Finally, 19 articles were selected for the meta-analysis. Fig 1 shows the study flow chart.

### Characteristics of included studies with quality assessment and publication bias

**Fig 2:**
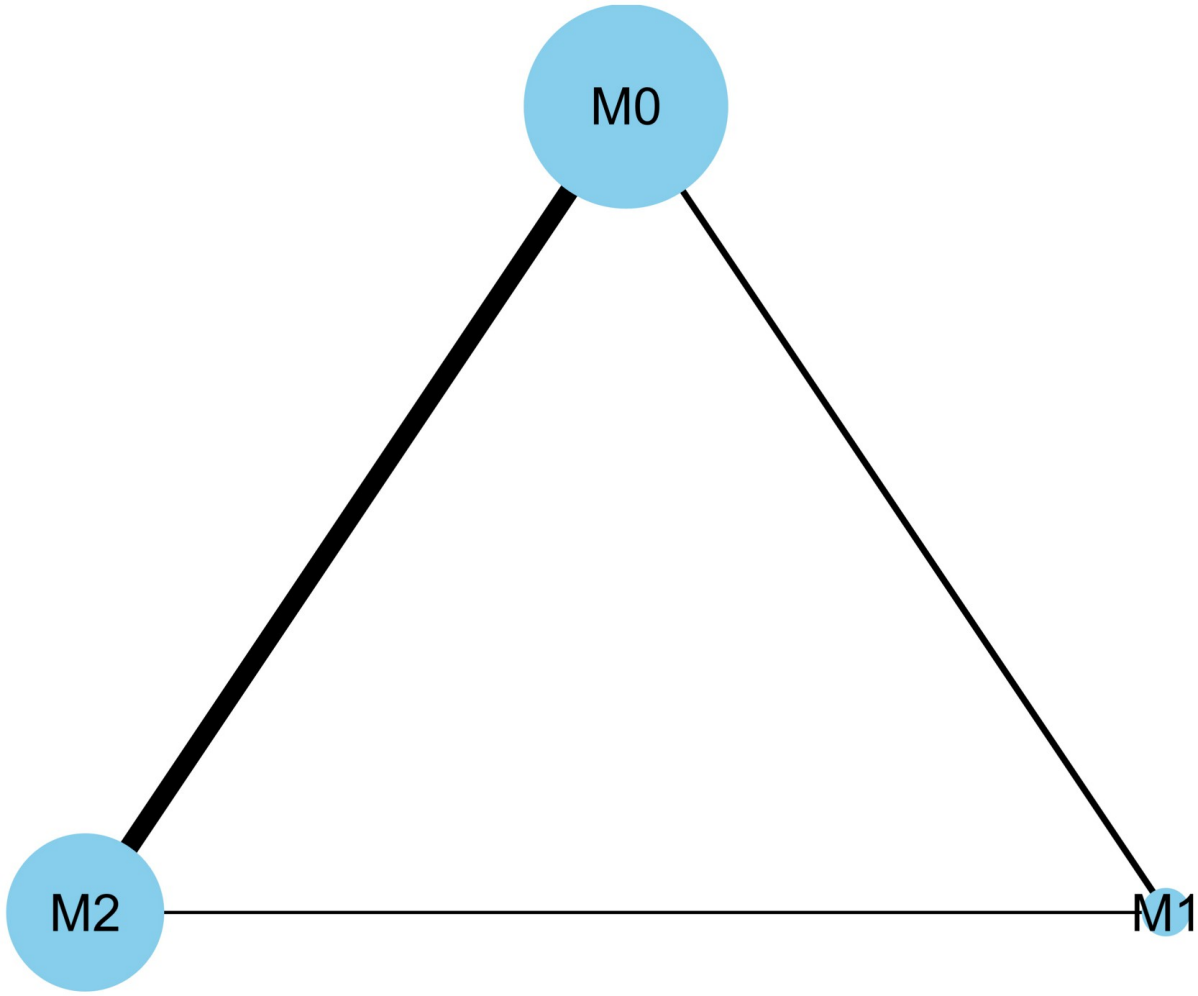
Network plots for included studies. There were just two published studies that included the M1 and M2 groups. Five studies included the M0 and M1 groups, and 12 studies included the M0 and M2 groups. M0, induction BCG therapy only; M1, 1-year BCG maintenance therapy; M2, BCG maintenance therapy for more than 1 year.

The characteristics and recurrence rates described in the 19 included studies are shown in Table 1 [13–31]. These eligible studies were published between 1987 and 2021. Nineteen studies with a total of 3,957 patients were included in the qualitative and quantitative analyses. There were just two published studies that included the M1 and M2 groups. Five studies included the M0 and M1 groups, and 12 studies included the M0 and M2 groups (Fig 2).

**Table 1:** Characteristics of included studies. M0, induction BCG therapy only; M1, 1-year BCG maintenance therapy; M2, BCG maintenance therapy for more than 1 year.

Category	Study	Methods	Study design	BCG strain	No. of Patients	Mean/median follow-up period	Recurrence	Quality assessment (SIGN)
M0 vs. M1	Mohamed et al. 2020 [13]	M0	Prospective	Pasteur	27	40 months	15	2+
		M1			26	35 months	5	
	Yoo et al. 2012 [14]	M0	Retrospective	OncoTICE	34	16.5 months	16^a^	1+
		M1			92	43 months	21^a^	
	Okamura et al. 2011 [15]	M0	Retrospective	Tokyo	27	66 months	13^b^	1+
		M1			48	102 months	8^b^	
	Koga et al. 2010 [16]	M0	Randomized controlled	Tokyo	27	28.7 months	7^a^	2+
		M1			24	26.5 months	1^a^	
	Akaza et al. 1995 [17]	M0	Randomized controlled	Tokyo	55	42 months	20	1+
		M1			52	48 months	22	
M0 vs. M2	Miyake et al. 2021 [18]	M0	Retrospective	Tokyo or Connaught	874	48 months	175^a^	2+
		M2			405		41^a^	
	Koguchi et al. 2020 [19]	M0	Retrospective	Tokyo	40	36.2 months	14	2+
		M2			38		5	
	Joshua et al. 2019 [20]	M0	Retrospective	Not addressed	40	Not addressed	8^a^	1+
		M2			61		7^a^	
	Yuk et al. 2018 [21]	M0	Retrospective	Not addressed	29	63 months	14	2+
		M2			26		5	
	Nakai et al. 2016 [22]	M0	Randomized controlled	Connaught	42	51 months	9^b^	2+
		M2			46		9^b^	
	Martínez-Piñeiro et al. 2015 [23]	M0	Randomized controlled	Connaught	195	103 months	80^b^	2+
		M2			202	102 months	68^b^	
	Muto et al. 2013 [24]	M0	Retrospective	Connaught	64	42.3±33.1 months	23^b^	1+
		M2			40	51.1±34.6 months	6^b^	
	Hinotsu et al. 2011 [25]	M0	Randomized controlled	Connaught	42	Not addressed	14^a^	1+
		M2			41		5^a^	
	Palou et al. 2001 [26]	M0	Randomized controlled	Connaught	61	77.8 months	16	2+
		M2			65		10	
	Lamm et al. 2000 [27]	M0	Randomized controlled	Connaught	192	Not addressed	113^b^	2+
		M2			192		77^b^	
	Badalament et al. 1987 [28]	M0	Randomized controlled	Pasteur	46	22 months	3	1+
		M2			47		6	
	Hudson et al. 1987 [29]	M0	Randomized controlled	Pasteur	21	17.2 months	6	1+
		M2			21		5	
M1 vs. M2	Gupta et al. 2020 [30]	M1	Randomized controlled	Moscow	38	Not addressed	5^b^	2+
		M2			40		6^b^	
	Oddens et al. 2013 [31]	M1	Randomized controlled	OncoTICE	339	7.1 years	145	2+
		M2			338		131	

^a^During the 2-year follow-up;

^b^during the 5-year follow-up.

The quality assessment was indicated by Scottish Intercollegiate Guidelines Network (SIGN) checklist. 1+ means well-conducted RCT with a low risk of bias. 1- means RCT with a high risk of bias. 2+ means well-conducted cohort studies with a low risk of bias. 2- means cohort studies with a high risk of bias.

**Fig 3:**
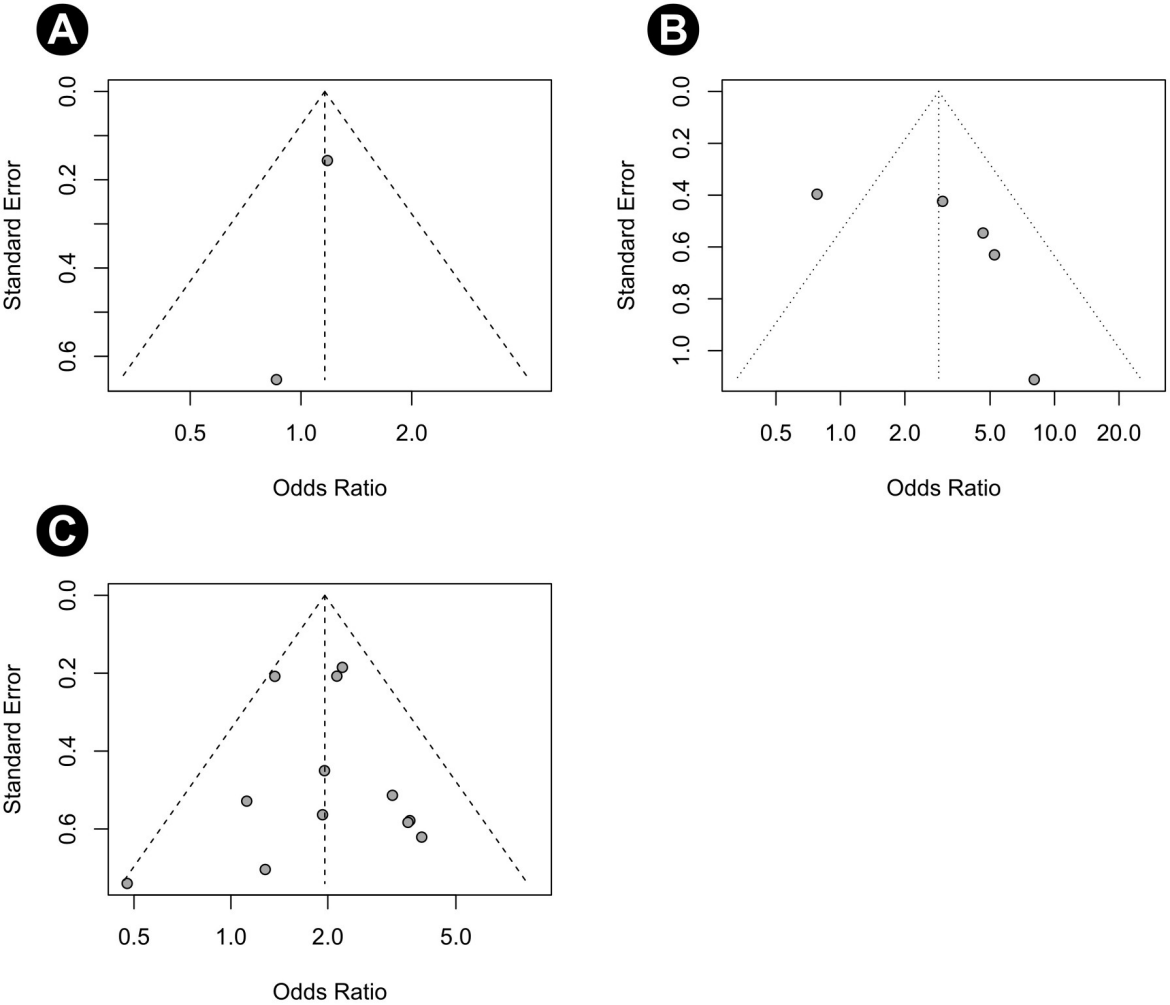
Funnel plot. (A) Recurrence rate in the M0 group, (B) recurrence rate in the M1 group, and (C) recurrence rate in the M2 group.

**Fig 4:**
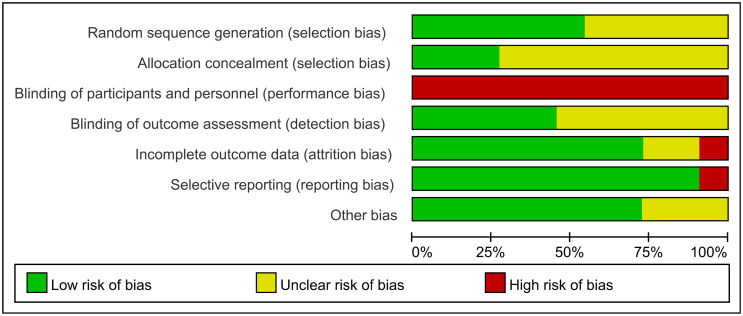
Risk of bias for eight RCTs. The risk of bias for each item is presented as a percentage across all included studies.

**Fig 5:**
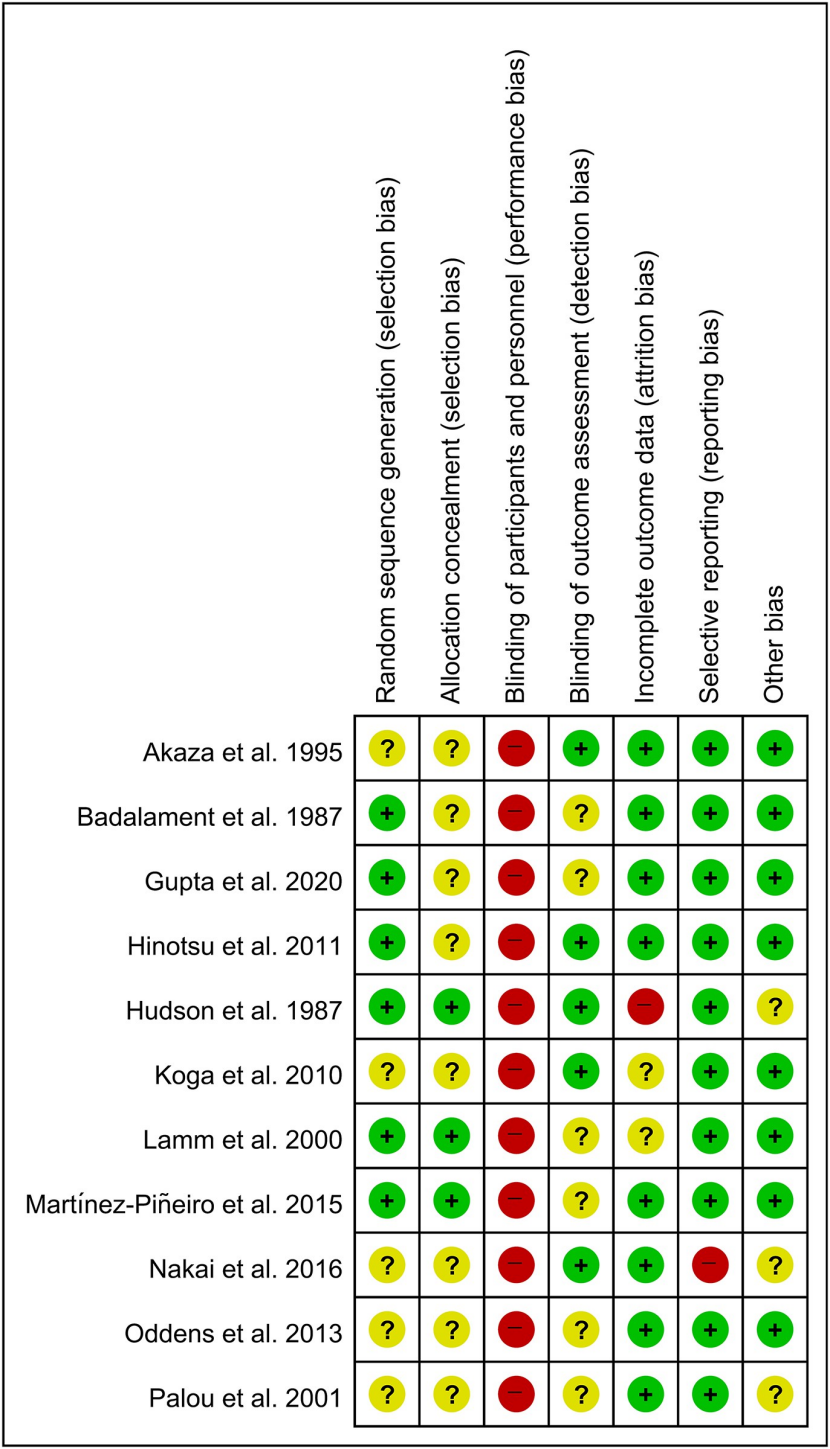
Risk of bias for eight RCTs. +, no bias;–, bias;?, bias unknown.

The quality assessment results using SIGN are provided in Table 1. Funnel plots of our study are shown in Fig 3. Most studies were located in the funnels. The risk of bias for eight RCTs is displayed in Figs 4 and 5. Adequate randomization methods and allocation concealment were described in only six and three studies, respectively. Blinding of outcome assessments was performed in five studies. The MINORS scores are shown in Table 2. All studies were considered appropriate.

**Table 2:** MINORS score in nonrandomized studies included in the review.

	A Clearly Stated Aim	Inclusion of Consecutive Samples	Prospective Collection of Data	Endpoints Appropriate to the Aim of the Study	Unbiased Assessment of the Study Endpoint	Follow-up Period Appropriate to the Aim of the Study	Loss to Follow-Up Less than 5%	Prospective Calculation of the Study Size	An Adequate Control Group	Contemporary Groups	Baseline Equivalence of Groups	Adequate Statistical Analyses	Total
Miyake et al. 2021	2	2	2	2	0	2	2	0	2	2	2	2	20
Mohamed et al. 2020	2	2	2	2	0	2	2	0	2	2	1	2	19
Koguchi et al. 2020	2	2	2	2	0	2	2	0	2	2	1	2	19
Joshua et al. 2019	2	2	2	2	0	2	2	0	2	2	1	2	19
Yuk et al. 2018	2	2	2	2	0	2	2	0	2	2	2	2	20
Muto et al. 2013	2	2	2	2	0	2	2	0	2	2	1	2	19
Yoo et al. 2012	2	2	2	2	0	2	2	0	2	2	2	2	20
Okamura et al. 2011	2	2	2	2	0	2	2	0	2	2	2	2	20

MINORS, methodological index for nonrandomized studies. The items are scored 0 (not reported), 1 (reported but inadequate), or 2 (reported and adequate). The global ideal score is 16 for non-comparative studies and 24 for comparative studies.

### Heterogeneity and inconsistency assessment

**Fig 6:**
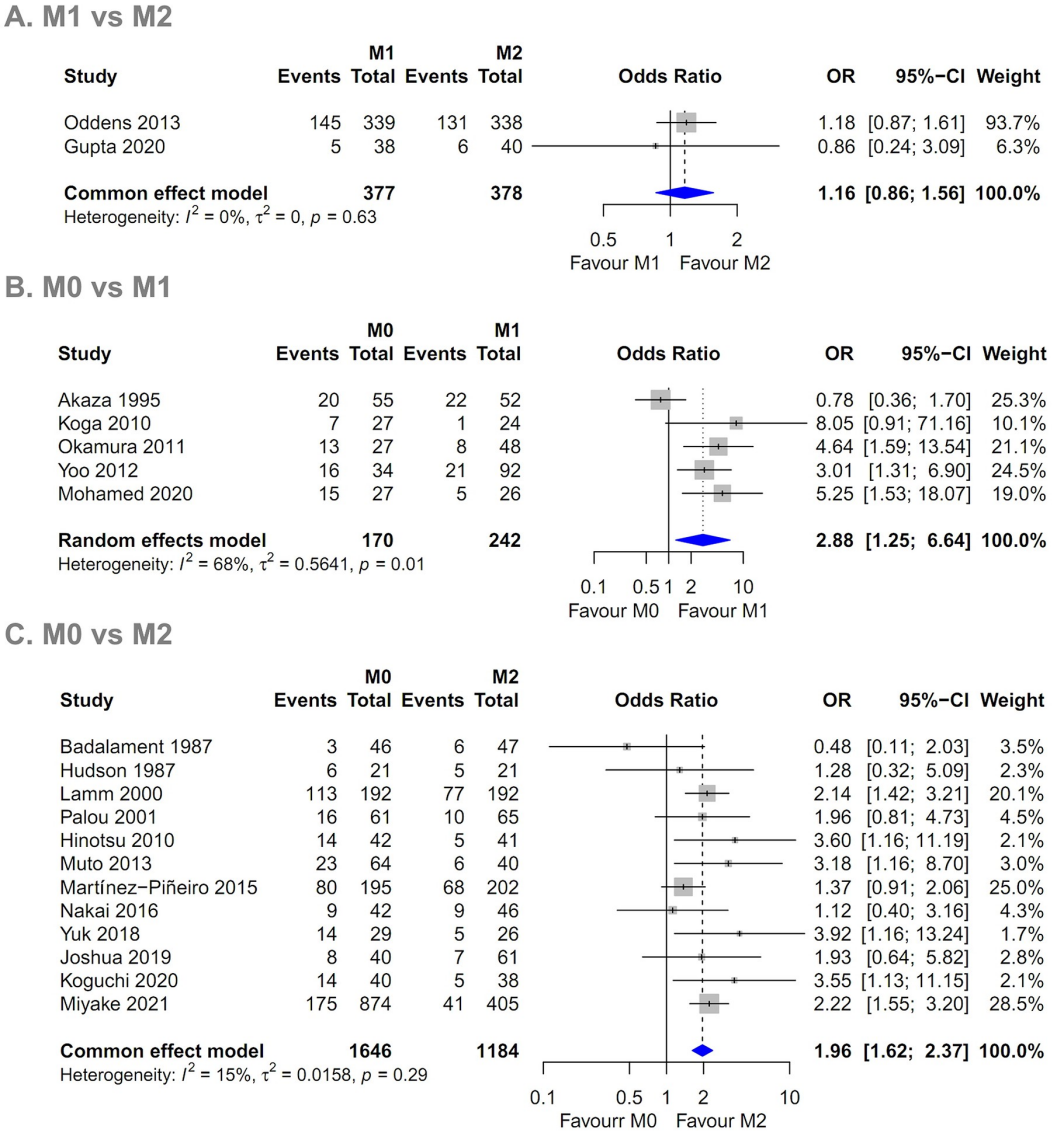
Pairwise meta-analysis of (A) M1 and M2 groups, (B) M0 and M1 groups, and (C) M0 and M2 groups. The recurrence rate in the M1 group was slightly higher than that in the M2 group (*P* = 0.328; OR 1.161, 95% CI 0.861–1.564). The recurrence rate in the M0 group was higher than that in the M1 (*P* = 0.013; OR 2.877, 95% CI 1.246–6.643) and M2 groups (*P* < 0.001; OR 1.958, 95% CI 1.618–2.369). M0, induction BCG therapy only; M1, 1-year BCG maintenance therapy; M2, BCG maintenance therapy for more than 1 year.

**Fig 7:**
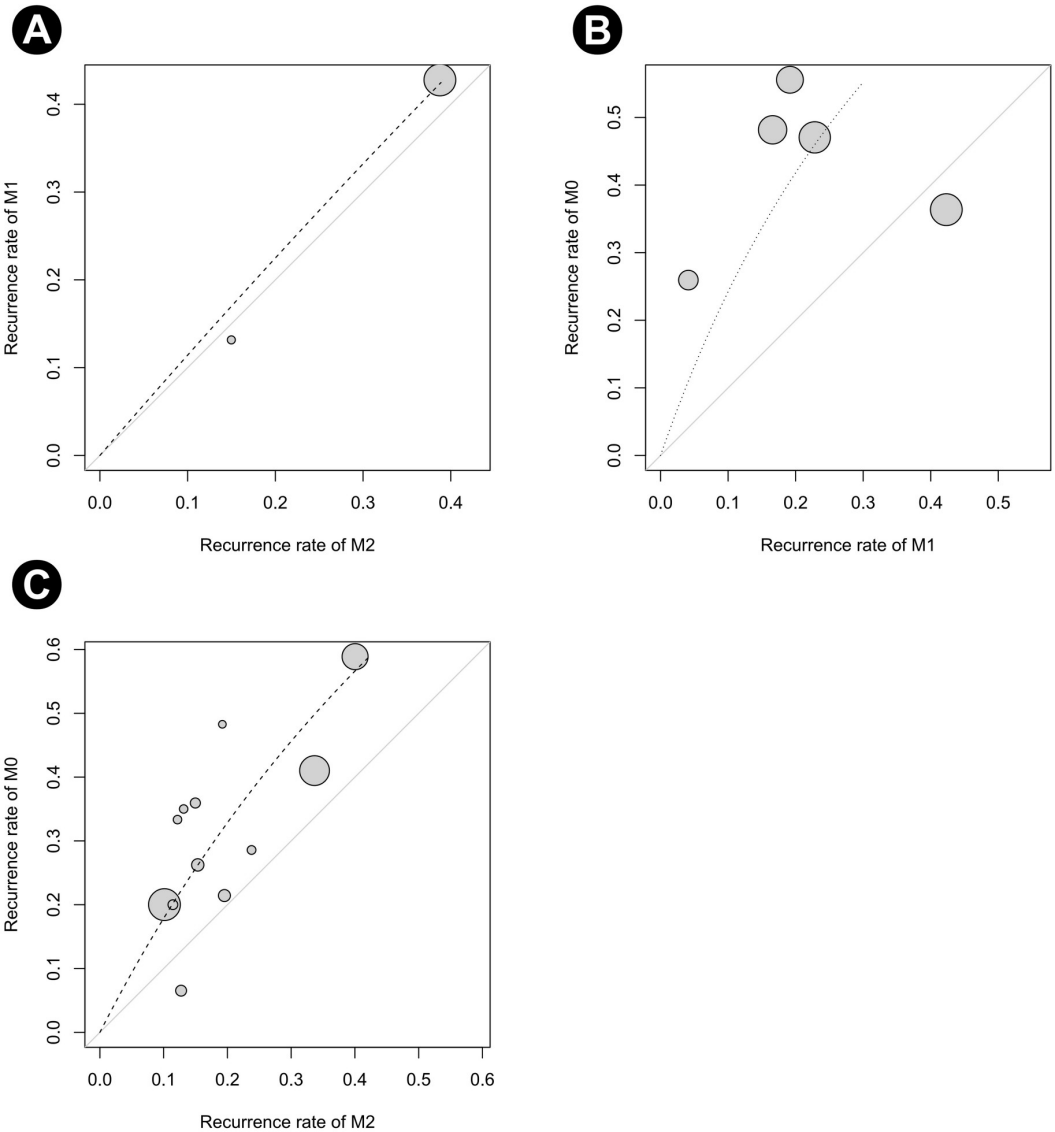
L’Abbe plots of recurrence rate. (A) Between the M1 and M2 groups, (B) between the M0 and M1 groups, and (C) between the M0 and M2 groups. M0, induction BCG therapy only; M1, 1-year BCG maintenance therapy; M2, BCG maintenance therapy for more than 1 year.

**Fig 8:**
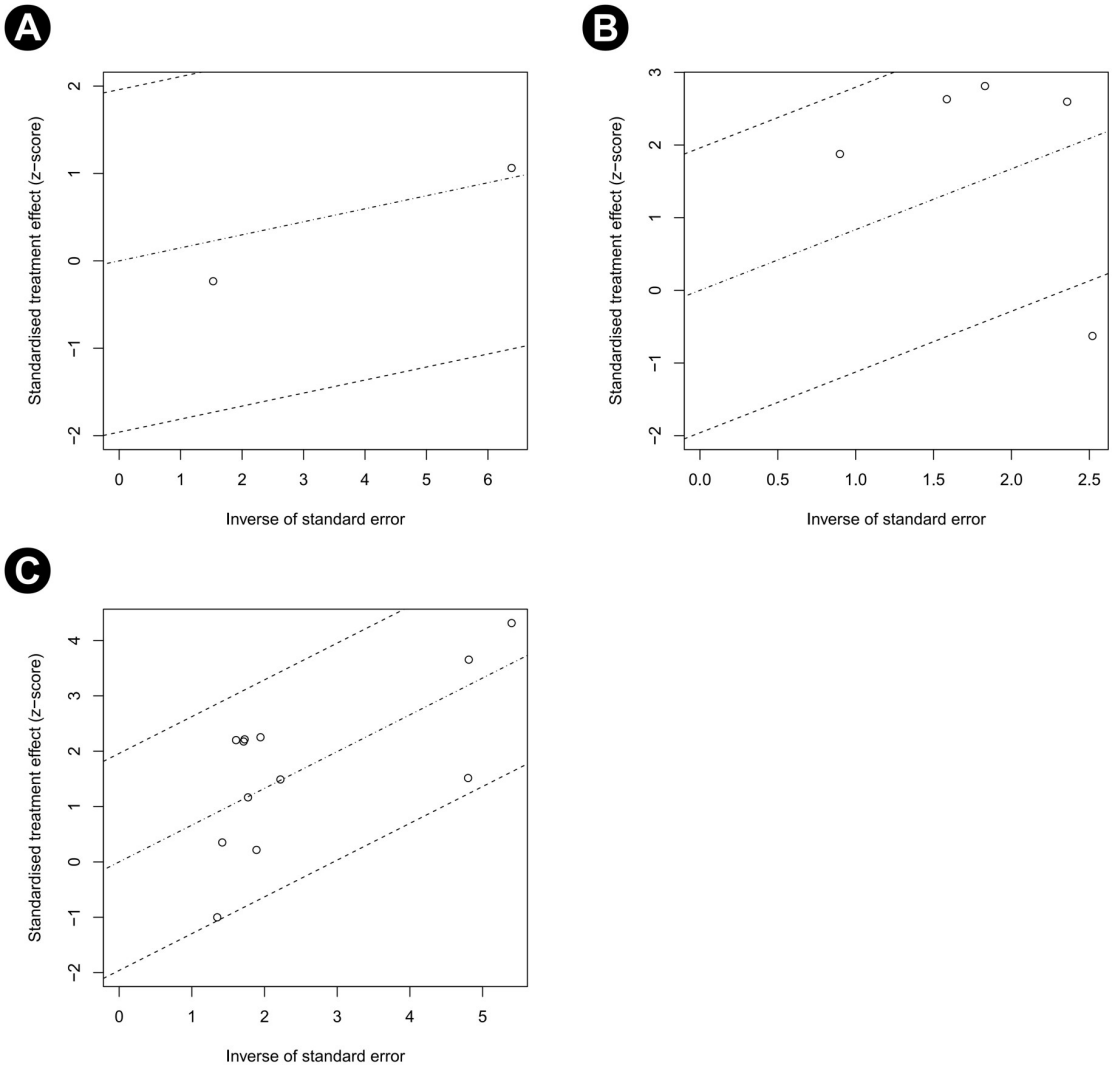
Radial plots of recurrence rate. (A) Between the M1 and M2 groups, (B) between the M0 and M1 groups, and (C) between the M0 and M2 groups. M0, induction BCG therapy only; M1, 1-year BCG maintenance therapy; M2, BCG maintenance therapy for more than 1 year.

**Fig 9:**
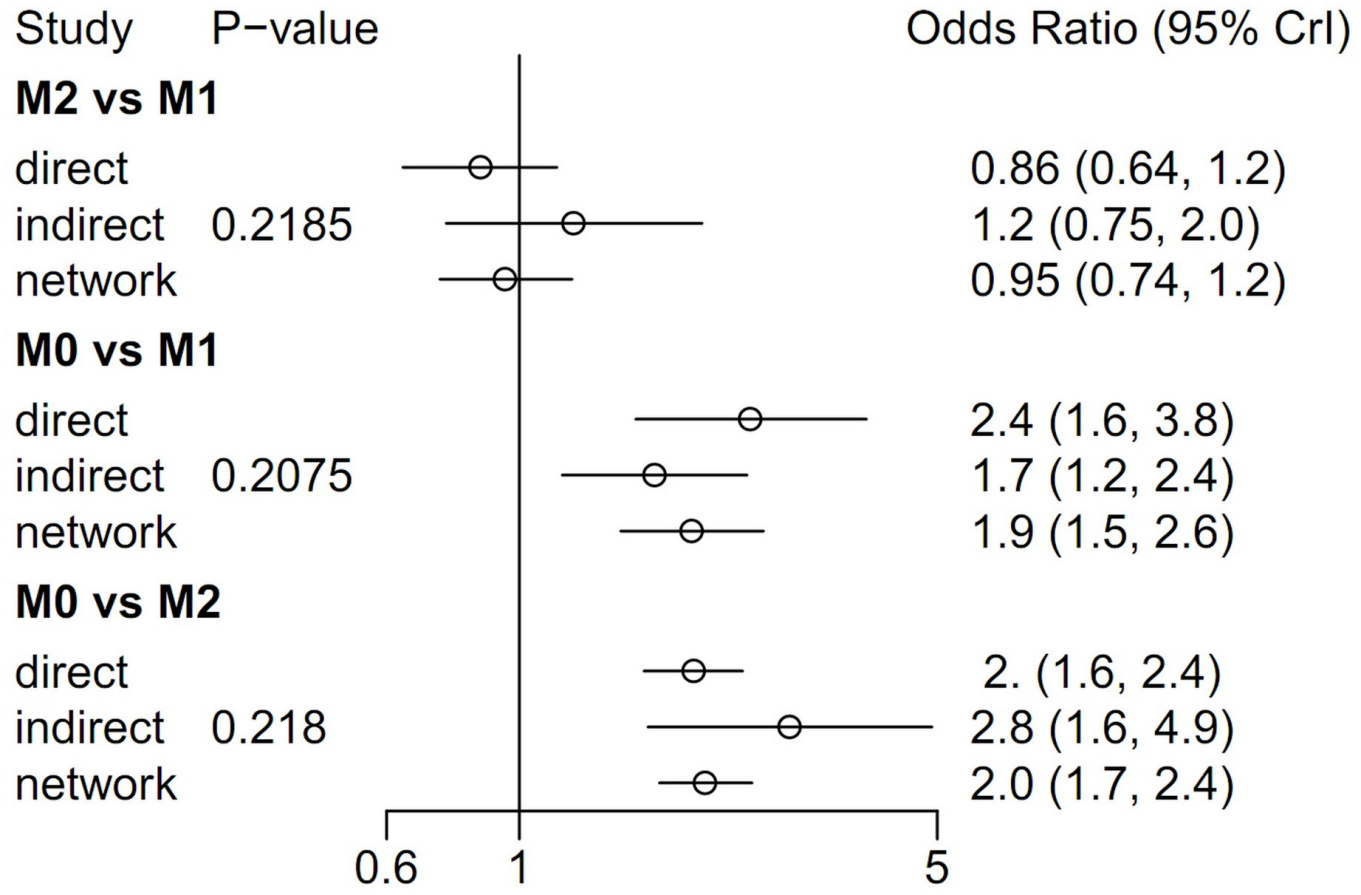
Node-split forest plot using MCMC modeling. M0, induction BCG therapy only; M1, 1-year BCG maintenance therapy; M2, BCG maintenance therapy for more than 1 year.

Forest plots of the pairwise meta-analysis results of the three groups are shown in Fig 6. There was no heterogeneity between groups M1 and M2 or between groups M0 and M2 in any study; however, there was little heterogeneity between groups M0 and M1. Therefore, a random-effects model was applied for a comparison of groups M0 and M1 (Fig 6B). After selection of the random-effects model, little heterogeneity was noted in L’Abbe plots (Fig 7) and radial plots (Fig 8). In the node-splitting analysis, no inconsistency was demonstrated in direct, indirect, or network comparisons (Fig 9).

### Pairwise meta-analysis of groups M0, M1, and M2

The recurrence rate of the M1 group was slightly higher than that of the M2 group (*P* = 0.328; OR 1.161, 95% CI 0.861–1.564) (Fig 6A). The recurrence rate of the M0 group was higher than that of the M1 group (*P* = 0.013; OR 2.877, 95% CI 1.246–6.643) (Fig 6B). The recurrence rate of the M0 group was also higher than that of the M2 group (*P* < 0.001; OR 1.958, 95% CI 1.618–2.369) (Fig 6C).

### Network meta-analysis of groups M0, M1, and M2 for recurrence rate

**Fig 10:**
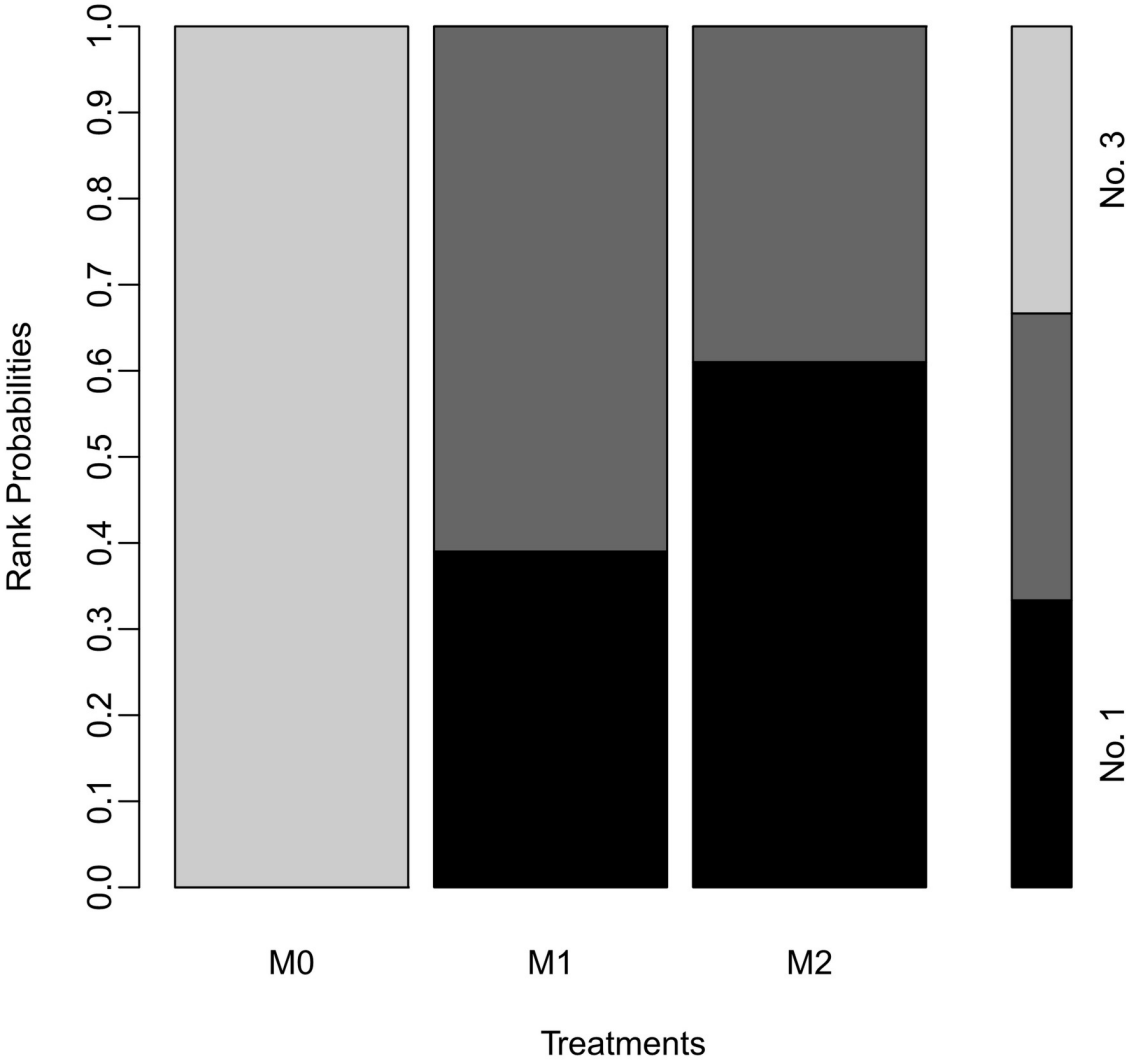
The rank-probability test using MCMC modeling. M0, induction BCG therapy only; M1, 1-year BCG maintenance therapy; M2, BCG maintenance therapy for more than 1 year.

In the node-split forest plot using Bayesian Markov Chain Monte Carlo (MCMC) modeling, there were no differences between the M1 and M2 groups in terms of recurrence rate [OR 0.95 (0.73–1.2)]. However, the recurrence rate in the M0 group was higher than those in the M1 [OR 1.9 (1.5–2.5)] and M2 [OR 2.0 (1.7–2.4)] groups (Fig 9). P-score tests using frequentist inference to rank treatments in the network demonstrated that the M2 group treatment (P-score 0.8701) was superior to the M1 (P-score 0.6299) and M0 groups (P-score 0). In the rank-probability test using MCMC modeling, the M2 group showed the highest rank, followed by the M1 and M0 groups (Fig 10).

## Discussion

BCG was first discovered by Albert Calmette and Camille Guerin as a tuberculosis vaccination in 1921 [32]. The first report of BCG as an immunotherapy for bladder cancer was published in 1959 [33]. In 1976, Morales et al. published a landmark paper about the beneficial effects of BCG immunotherapy on recurrent superficial bladder cancer [34]. In 1980, the first controlled trial showing similar results was published, and in 1990, BCG received Food and Drug Administration approval for the treatment of superficial bladder cancer [35].

Several studies have demonstrated that BCG maintenance treatments show clinical benefit in patients with high-risk NMIBC. Lamm et al. reported the effectiveness of BCG maintenance treatments in 384 patients with recurrent NMIBC [27]. Compared with standard induction BCG immunotherapy, BCG maintenance immunotherapy was favorable in patients with superficial bladder cancer. Compared to the induction therapy-only arm, patients in the 3-week maintenance arm showed twice as long median recurrence-free survival (RFS) and significantly longer progression-free survival. Mohamed et al. reported a prospective randomized study comparing 31 patients with NMIBC who underwent induction therapy only and 35 patients with NMIBC who underwent induction therapy plus a 1-year maintenance therapy [13]. Patients who received only induction therapy had significantly higher recurrence rates than those who received maintenance therapy. The 5-year RFS rate was 41% in the induction therapy group and 78% in the maintenance therapy group. To date, BCG maintenance treatments are considered to exert clinical benefits, especially in terms of preventing recurrence of NMIBC.

Although the optimal duration of maintenance therapy for patients with high-risk NMIBC remains controversial, EAU’s guidelines recommend 3-week instillations at 3, 6, 12, 18, 24, 30, and 36 months. As the COVID-19 pandemic has had a serious impact on urological treatments [9], Lenfant et al. reported that intravesical BCG therapy could be discontinued safely for patients with high-risk NMIBC [8]. Furthermore, according to EAU’s NMIBC panel during the pandemic, a 1-year BCG maintenance immunotherapy in patients with high-risk NMIBC was recommended [10].

There have been several systematic reviews and meta-analyses on intravesical BCG treatment in patients with NMIBC. Chen et al. conducted a systematic review and meta-analysis of 10 RCTs [36], showing that BCG maintenance therapy could decrease the risk of tumor recurrence by 21% and prolong RFS by 33% compared with nonmaintenance therapy. In addition, they showed that BCG maintenance therapy could decrease the risk of tumor progression. These results are the basis for the effectiveness of BCG maintenance treatment. Quan et al. conducted a study about dose, duration, and BCG strain for the treatment of patients with NMIBC [4]. Finally, 19 studies were selected for a meta-analysis. Low-dose BCG and induction therapy-only groups showed significantly higher risks of recurrence [risk ratio (RR) 1.17 and 1.33, respectively]. These results may serve as the basis for the better clinical outcomes of the 3-year maintenance therapy. Huang et al. conducted a systematic review and meta-analysis of nine RCTs [37]. Similar to our study results, longer BCG maintenance therapy (such as 3 years) did not significantly reduce the risk of tumor recurrence or progression compared to shorter-term BCG maintenance therapy (such as 1 year). However, the limitation is that all of these studies were conducted prior to the COVID-19 pandemic.

The characteristics of patients with NMIBC are generally a high median age of 70 years, many comorbidities, and a high smoking rate, which all increase the risk of COVID-19 severity [38]. Our study was conducted in consideration of these risks and EAU’s COVID-19 recommendations; in the short term, there was no significant difference between maintenance therapy groups. Gallegos et al. conducted a prospective study on a total of 175 patients with NMIBC who received BCG treatment at a Chilean hospital from 2019 to 2020 [39]. Throughout the study duration, 43 patients were diagnosed with COVID-19. In these patients, only one patient died from the disease (case fatality rate = 2.3%) during follow-up. They also compared patients with COVID-19 receiving BCG treatment with the overall population of the same age (70–79 years), according to the Chilean national register. During the same follow-up duration, 6.3% of the control group became infected with COVID-19, with a 14% case fatality rate. With regard to the study results, patients with NMIBC receiving the BCG immunotherapy showed a lower case fatality rate than the control group, but a higher rate of COVID-19 infection. The cause of the high rate of infection in the BCG treatment group is unclear. However, since social distancing must be maintained to prevent COVID-19 infection, reducing the duration of maintenance treatment according to EAU’s COVID-19 recommendations is a reasonable approach to take during the COVID-19 pandemic.

During the COVID-19 pandemic, management also has been delayed for muscle-invasive bladder cancer (MIBC). According to EAU recommendations, Kang et al. conducted systematic review and meta-analysis to evaluate the efficacy of neoadjuvant chemotherapy (NAC) compared with radical cystectomy (RC) alone in improving the overall survival (OS) of patients with T2-4aN0M0 MIBC [40]. The OS was significantly better in the NAC with RC group than in the RC alone group. However, in a subgroup analysis of patients with T2N0M0 MIBC, there was no difference in the OS between the NAC with RC group and the RC alone group. They concluded that, as recommended by the EAU Guidelines Office Rapid Reaction Group, patients with T2N0M0 MIBC should strongly consider omitting NAC until the end of the COVID-19 pandemic.

BCG unresponsiveness is one of the important considerations in BCG therapy for patients with NMIBC. According to the EAU guidelines on NMIBC, BCG unresponsive tumors included all BCG refractory tumors (T1G3/high-grade (HG) tumor at 3 months; TaG3/HG tumor after 3 months and/or at 6 months, after either re-induction or first course of maintenance; carcinoma in situ (CIS), without concomitant papillary tumor, at 3 months and persisting at 6 months after either re-induction or first course of maintenance; HG tumor during BCG maintenance therapy) and those who develop T1Ta/HG recurrence within 6 months or CIS within 12 months from the completion of adequate BCG exposure [41]. In general, BCG-unresponsive patients have worse oncological outcomes, and therefore, studies related to factors that can predict BCG response are important in the treatment of NMIBC. Ferro *et al*. conducted a retrospective study to investigate the predictive factors in the response to BCG in patients with a T1G3/HG NMIBC diagnosis [42]. According to their study, multifocality, lymphovascular invasion, and HG on re-TURBT were independent predictors for response to BCG treatment. To reduce the risk of understaging and missing MIBC, re-TURBT should be performed, especially in HG NMIBC. According to another study, independent predictors to identify patients at risk of residual HG disease after a complete TURBT include the tumor size, presence of CIS, and body mass index (BMI)≥25 kg/m^2^ [43]. When deciding to perform re-TURBT, the presence of these factors would also be an important consideration.

The main strength of our study is that it is the first to evaluate differences in clinical outcomes with regard to the period of intravesical BCG maintenance therapy for patients with NMIBC during the era of COVID-19. Considering the COVID-19 infection rate, the fatality rate in patients with NMIBC, and the increase in the number of COVID-19-related deaths, it is reasonable to follow EAU’s COVID-19 recommendations in the post-COVID-19 era. However, if COVID-19 becomes a controllable disease, a conventional BCG maintenance therapy can help decrease the recurrence rate of NMIBC. According to EAU’s COVID-19 recommendations, studies on patients receiving BCG maintenance therapy for 1 year should be conducted in the near future, and preparations for another pandemic should be made.

The main limitation of our meta-analysis is its scant heterogeneity across the studies in terms of different treatment regimens and different strains of BCG used. Second, RCTs and non-RCT studies were mixed and analyzed in our network meta-analysis. Third, other clinical outcomes, such as progression rate and survival rate, were not analyzed. Further analysis of progression and survival rates is expected to increase the reliability of our results. Fourth, the side effects of intravesical BCG maintenance therapy and subsequent treatment tolerance were not analyzed. The severity of side effects is a significant factor in deciding on the duration of intravesical BCG therapy.

## Conclusions

In our network meta-analysis, there was no difference between BCG maintenance therapy groups in terms of recurrence rate. In the rank test, BCG therapy lasting more than 1-year appears to be most effective in patients with NMIBC.

Given the COVID-19 infection rate, fatality rate of NMIBC, and increase in the number of COVID-19 deaths, it is reasonable to follow the EAU COVID-19 recommendation for the post-COVID-19 era. However, if COVID-19 becomes a controllable disease, conventional BCG maintenance therapy might help decrease the recurrence rate of NMIBC. Studies of patients receiving 1-year maintenance therapy should be conducted in the near future, and preparations for another pandemic should be made.

## Supporting information

S1 Table PRISMA checklist. (DOCX)

## References

[1] Saginala K, Barsouk A, Aluru JS, Rawla P, Padala SA, Barsouk A. Epidemiology of Bladder Cancer. Med Sci (Basel). 2020;8(1). Epub 2020/03/19. doi: 10.3390/medsci8010015 . 32183076 PMC7151633

[2] Gan C, Mostafid H, Khan MS, Lewis DJ. BCG immunotherapy for bladder cancer—the effects of substrain differences. Nat Rev Urol. 2013;10(10):580–8. Epub 2013/09/18. doi: 10.1038/nrurol.2013.194 . 24042563

[3] Thyavihally YB, Dev P, Waigankar S, Pednekar A, Athikari N, Raut A, et al. Intravesical bacillus Calmette-Guerin (BCG) in treating non-muscle invasive bladder cancer—analysis of adverse effects and effectiveness of two strains of BCG (Danish 1331 and Moscow). Asian Journal of Urology. 2021. doi: 10.1016/j.ajur.2021.05.002 35509489 PMC9051354

[4] Quan Y, Jeong CW, Kwak C, Kim HH, Kim HS, Ku JH. Dose, duration and strain of bacillus Calmette-Guerin in the treatment of nonmuscle invasive bladder cancer: Meta-analysis of randomized clinical trials. Medicine (Baltimore). 2017;96(42):e8300. Epub 2017/10/20. doi: 10.1097/MD.0000000000008300 . 29049231 PMC5662397

[5] Babjuk M, Burger M, Compérat EM, Gontero P, Mostafid AH, Palou J, et al. European Association of Urology Guidelines on Non-muscle-invasive Bladder Cancer (TaT1 and Carcinoma In Situ)—2019 Update. Eur Urol. 2019;76(5):639–57. Epub 2019/08/25. doi: 10.1016/j.eururo.2019.08.016 . 31443960

[6] Fuge O, Vasdev N, Allchorne P, Green JS. Immunotherapy for bladder cancer. Res Rep Urol. 2015;7:65–79. Epub 2015/05/23. doi: 10.2147/RRU.S63447 . 26000263 PMC4427258

[7] Kapoor R, Vijjan V, Singh P. Bacillus Calmette-Guérin in the management of superficial bladder cancer. Indian J Urol. 2008;24(1):72–6. Epub 2008/01/01. doi: 10.4103/0970-1591.38608 . 19468364 PMC2684253

[8] Lenfant L, Seisen T, Loriot Y, Rouprêt M. Adjustments in the Use of Intravesical Instillations of Bacillus Calmette-Guérin for High-risk Non-muscle-invasive Bladder Cancer During the COVID-19 Pandemic. Eur Urol. 2020;78(1):1–3. Epub 2020/05/01. doi: 10.1016/j.eururo.2020.04.039 . 32349928 PMC7180366

[9] Moussa M, Chakra MA, Papatsoris AG, Dellis A. The Impact of COVID-19 Disease on Urology Practice. Surg J (N Y). 2021;7(2):e83–e91. Epub 2021/06/10. doi: 10.1055/s-0041-1725155 . 34104720 PMC8175122

[10] Ribal MJ, Cornford P, Briganti A, Knoll T, Gravas S, Babjuk M, et al. European Association of Urology Guidelines Office Rapid Reaction Group: An Organisation-wide Collaborative Effort to Adapt the European Association of Urology Guidelines Recommendations to the Coronavirus Disease 2019 Era. Eur Urol. 2020;78(1):21–8. Epub 2020/05/08. doi: 10.1016/j.eururo.2020.04.056 . 32376137 PMC7183974

[11] Moher D, Liberati A, Tetzlaff J, Altman DG. Preferred reporting items for systematic reviews and meta-analyses: the PRISMA statement. Bmj. 2009;339:b2535. Epub 2009/07/23. doi: 10.1136/bmj.b2535 . 19622551 PMC2714657

[12] Moher D, Liberati A, Tetzlaff J, Altman DG. Preferred reporting items for systematic reviews and meta-analyses: the PRISMA statement. PLoS Med. 2009;6(7):e1000097. Epub 2009/07/22. doi: 10.1371/journal.pmed.1000097 . 19621072 PMC2707599

[13] Mohamed MB, Ali MH, Shamaa MA, Shaaban SM. Single course of intravesical Bacillus Calmette-Guerin versus single course with maintenance therapy in the management of nonmuscle invasive bladder cancer: A prospective randomized study. Urol Ann. 2020;12(4):360–5. Epub 2021/03/30. doi: 10.4103/UA.UA_137_19 . 33776333 PMC7992517

[14] Yoo KH, Lim TJ, Chang SG. Monthly intravesical bacillus Calmette-Guérin maintenance therapy for non-muscle-invasive bladder cancer: 10-year experience in a single institute. Exp Ther Med. 2012;3(2):221–5. Epub 2012/09/13. doi: 10.3892/etm.2011.400 . 22969872 PMC3438621

[15] Okamura T, Akita H, Ando R, Ikegami Y, Naiki T, Kawai N, et al. Single monthly bacillus Calmette-Guérin intravesical instillation is effective maintenance therapy to prevent recurrence in Japanese patients with non-muscle-invasive bladder cancer. Int J Clin Oncol. 2012;17(5):477–81. Epub 2011/09/29. doi: 10.1007/s10147-011-0314-3 . 21947596

[16] Koga H, Ozono S, Tsushima T, Tomita K, Horiguchi Y, Usami M, et al. Maintenance intravesical bacillus Calmette-Guérin instillation for Ta, T1 cancer and carcinoma in situ of the bladder: randomized controlled trial by the BCG Tokyo Strain Study Group. Int J Urol. 2010;17(9):759–66. Epub 2010/07/08. doi: 10.1111/j.1442-2042.2010.02584.x . 20604814

[17] Akaza H, Hinotsu S, Aso Y, Kakizoe T, Koiso K. Bacillus Calmette-Guérin treatment of existing papillary bladder cancer and carcinoma in situ of the bladder. Four-year results. The Bladder Cancer BCG Study Group. Cancer. 1995;75(2):552–9. Epub 1995/01/15. doi: 10.1002/1097-0142(19950115)75:2&lt;552::aid-cncr2820750219&gt;3.0.co;2-h . 7812924

[18] Miyake M, Iida K, Nishimura N, Miyamoto T, Fujimoto K, Tomida R, et al. Non-maintenance intravesical Bacillus Calmette-Guérin induction therapy with eight doses in patients with high- or highest-risk non-muscle invasive bladder cancer: a retrospective non-randomized comparative study. BMC Cancer. 2021;21(1):266. Epub 2021/03/13. doi: 10.1186/s12885-021-07966-7 . 33706705 PMC7948348

[19] Koguchi D, Matsumoto K, Hirayama T, Moroo S, Kobayashi M, Katsumata H, et al. Impact of maintenance therapy using a half dose of the bacillus Calmette-Guérin Tokyo strain on recurrence of intermediate and high-risk nonmuscle invasive bladder cancer: a retrospective single-center study. BMC Urol. 2020;20(1):194. Epub 2020/12/11. doi: 10.1186/s12894-020-00766-4 . 33298034 PMC7726881

[20] Joshua JM, Vijayan M, Pooleri GK. A retrospective analysis of patients treated with intravesical BCG for high-risk nonmuscle invasive bladder cancer. Ther Adv Urol. 2019;11:1756287219833056. Epub 2019/03/13. doi: 10.1177/1756287219833056 . 30858894 PMC6402057

[21] Yuk HD, Jeong CW, Kwak C, Kim HH, Ku JH. Should intravesical Bacillus Calmette-Guerin (BCG) treatment be administered to patients with T0 after repeat transurethral resection of bladder tumor in patients with high-risk non-muscle invasive bladder cancer? PLoS One. 2018;13(11):e0208267. Epub 2018/11/30. doi: 10.1371/journal.pone.0208267 . 30496283 PMC6264801

[22] Nakai Y, Anai S, Tanaka N, Chihara Y, Haramoto M, Otani T, et al. Insignificant role of bacillus Calmette-Guérin maintenance therapy after complete transurethral resection of bladder tumor for intermediate- and high-risk non-muscle-invasive bladder cancer: Results from a randomized trial. Int J Urol. 2016;23(10):854–60. Epub 2016/07/16. doi: 10.1111/iju.13167 . 27416975

[23] Martínez-Piñeiro L, Portillo JA, Fernández JM, Zabala JA, Cadierno I, Moyano JL, et al. Maintenance Therapy with 3-monthly Bacillus Calmette-Guérin for 3 Years is Not Superior to Standard Induction Therapy in High-risk Non-muscle-invasive Urothelial Bladder Carcinoma: Final Results of Randomised CUETO Study 98013. Eur Urol. 2015;68(2):256–62. Epub 2015/03/22. doi: 10.1016/j.eururo.2015.02.040 . 25794457

[24] Muto S, Nakajima A, Horiuchi A, Inoue M, China T, Saito K, et al. Maintenance therapy with intravesical bacillus Calmette-Guerin in patients with intermediate- or high-risk non-muscle-invasive bladder cancer. Jpn J Clin Oncol. 2013;43(3):305–13. Epub 2013/01/11. doi: 10.1093/jjco/hys225 . 23303841

[25] Hinotsu S, Akaza H, Naito S, Ozono S, Sumiyoshi Y, Noguchi S, et al. Maintenance therapy with bacillus Calmette-Guérin Connaught strain clearly prolongs recurrence-free survival following transurethral resection of bladder tumour for non-muscle-invasive bladder cancer. BJU Int. 2011;108(2):187–95. Epub 2010/12/24. doi: 10.1111/j.1464-410X.2010.09891.x . 21176079

[26] Palou J, Laguna P, Millán-Rodríguez F, Hall RR, Salvador-Bayarri J, Vicente-Rodríguez J. Control group and maintenance treatment with bacillus Calmette-Guerin for carcinoma in situ and/or high grade bladder tumors. J Urol. 2001;165(5):1488–91. Epub 2001/05/09. . 11342902

[27] Lamm DL, Blumenstein BA, Crissman JD, Montie JE, Gottesman JE, Lowe BA, et al. Maintenance bacillus Calmette-Guerin immunotherapy for recurrent TA, T1 and carcinoma in situ transitional cell carcinoma of the bladder: a randomized Southwest Oncology Group Study. J Urol. 2000;163(4):1124–9. Epub 2000/03/29. . 10737480

[28] Badalament RA, Herr HW, Wong GY, Gnecco C, Pinsky CM, Whitmore WF Jr., et al. A prospective randomized trial of maintenance versus nonmaintenance intravesical bacillus Calmette-Guérin therapy of superficial bladder cancer. J Clin Oncol. 1987;5(3):441–9. Epub 1987/03/01. doi: 10.1200/JCO.1987.5.3.441 . 3546618

[29] Hudson MA, Ratliff TL, Gillen DP, Haaff EO, Dresner SM, Catalona WJ. Single course versus maintenance bacillus Calmette-Guerin therapy for superficial bladder tumors: a prospective, randomized trial. J Urol. 1987;138(2):295–8. Epub 1987/08/01. doi: 10.1016/s0022-5347(17)43125-9 . 3298694

[30] Gupta NK, Sarkar D, Pal DK. Monthly maintenance protocol Bacillus Calmette-Guerin as a viable alternative to Southwest Oncology Group maintenance protocol in nonmuscle-invasive bladder cancer: A prospective randomized study. Urol Ann. 2020;12(2):116–21. Epub 2020/06/23. doi: 10.4103/UA.UA_29_19 . 32565647 PMC7292436

[31] Oddens J, Brausi M, Sylvester R, Bono A, van de Beek C, van Andel G, et al. Final results of an EORTC-GU cancers group randomized study of maintenance bacillus Calmette-Guérin in intermediate- and high-risk Ta, T1 papillary carcinoma of the urinary bladder: one-third dose versus full dose and 1 year versus 3 years of maintenance. Eur Urol. 2013;63(3):462–72. Epub 2012/11/13. doi: 10.1016/j.eururo.2012.10.039 . 23141049

[32] Kamat AM, Flaig TW, Grossman HB, Konety B, Lamm D, O’Donnell MA, et al. Expert consensus document: Consensus statement on best practice management regarding the use of intravesical immunotherapy with BCG for bladder cancer. Nat Rev Urol. 2015;12(4):225–35. Epub 2015/03/25. doi: 10.1038/nrurol.2015.58 . 25800393

[33] Old LJ, Clarke DA, Benacerraf B. Effect of Bacillus Calmette-Guerin infection on transplanted tumours in the mouse. Nature. 1959;184(Suppl 5):291–2. Epub 1959/07/25. doi: 10.1038/184291a0 . 14428599

[34] Morales A, Eidinger D, Bruce AW. Intracavitary Bacillus Calmette-Guerin in the treatment of superficial bladder tumors. J Urol. 1976;116(2):180–3. Epub 1976/08/01. doi: 10.1016/s0022-5347(17)58737-6 . 820877

[35] Lamm DL, Thor DE, Harris SC, Reyna JA, Stogdill VD, Radwin HM. Bacillus Calmette-Guerin immunotherapy of superficial bladder cancer. J Urol. 1980;124(1):38–40. Epub 1980/07/01. doi: 10.1016/s0022-5347(17)55282-9 . 6997513

[36] Chen S, Zhang N, Shao J, Wang X. Maintenance versus non-maintenance intravesical Bacillus Calmette-Guerin instillation for non-muscle invasive bladder cancer: A systematic review and meta-analysis of randomized clinical trials. Int J Surg. 2018;52:248–57. Epub 2018/03/03. doi: 10.1016/j.ijsu.2018.02.045 . 29499363

[37] Huang Z, Liu H, Wang Y, Zhang C, Xu T. Determining optimal maintenance schedules for adjuvant intravesical bacillus Calmette-Guerin immunotherapy in non-muscle-invasive bladder cancer: a systematic review and network meta-analysis. Curr Med Res Opin. 2017;33(8):1379–87. Epub 2017/05/05. doi: 10.1080/03007995.2017.1326889 . 28471272

[38] Mossanen M. The Epidemiology of Bladder Cancer. Hematol Oncol Clin North Am. 2021;35(3):445–55. Epub 2021/05/08. doi: 10.1016/j.hoc.2021.02.001 . 33958144

[39] Gallegos H, Rojas PA, Sepúlveda F, Zúñiga Á, San Francisco IF. Protective role of intravesical BCG in COVID-19 severity. BMC Urol. 2021;21(1):50. Epub 2021/04/01. doi: 10.1186/s12894-021-00823-6 . 33785004 PMC8007648

[40] Kang DH, Cho KS, Moon YJ, Chung DY, Jung HD, Lee JY. Effect of neoadjuvant chemotherapy on overall survival of patients with T2-4aN0M0 bladder cancer: A systematic review and meta-analysis according to EAU COVID-19 recommendation. PLoS One. 2022;17(4):e0267410. Epub 2022/04/22. doi: 10.1371/journal.pone.0267410 . 35446917 PMC9022827

[41] Babjuk M, Burger M, Capoun O, Cohen D, Compérat EM, Dominguez Escrig JL, et al. European Association of Urology Guidelines on Non-muscle-invasive Bladder Cancer (Ta, T1, and Carcinoma in Situ). Eur Urol. 2022;81(1):75–94. Epub 2021/09/14. doi: 10.1016/j.eururo.2021.08.010 . 34511303

[42] Ferro M, Barone B, Crocetto F, Lucarelli G, Busetto GM, Del Giudice F, et al. Predictive clinico-pathological factors to identify BCG, unresponsive patients, after re-resection for T1 high grade non-muscle invasive bladder cancer. Urol Oncol. 2022. Epub 2022/06/09. doi: 10.1016/j.urolonc.2022.05.016 . 35676172

[43] Ferro M, Di Lorenzo G, Buonerba C, Lucarelli G, Russo GI, Cantiello F, et al. Predictors of Residual T1 High Grade on Re-Transurethral Resection in a Large Multi-Institutional Cohort of Patients with Primary T1 High-Grade/Grade 3 Bladder Cancer. J Cancer. 2018;9(22):4250–4. Epub 2018/12/07. doi: 10.7150/jca.26129 . 30519326 PMC6277616

